# Harmonizing Data Collection and Analysis In Worldwide FINGERS Trials

**DOI:** 10.1093/geroni/igab046.261

**Published:** 2021-12-17

**Authors:** Markku Peltonen

**Affiliations:** Population Health Unit, Helsinki, Uusimaa, Finland

## Abstract

One of the goals of the World-Wide FINGERS (WW-FINGERS): A Global Approach to Dementia Prevention network is to prospectively facilitate data sharing and joint analyses across clinical trials on prevention of cognitive impairment and dementia. The aim with prospectively harmonizing studies in different countries and settings regarding interventions, outcomes, measurements, data collection, and establishing best practices for responsible data sharing and access to data for remote joint analyses, is to increase the use of clinical trial data. By utilizing federated database system which connects multiple autonomous, decentralized databases and enables data-analyses to be conducted without individual level data being transferred, could be a feasible and acceptable technical solution for countries around the world, given wide variations in data protection and sharing regulations. Ultimately, prospectively harmonizing clinical studies and establishing a culture of harmonization and sharing, will promote international joint initiatives to identify globally implementable and effective preventive strategies.

